# Supplementary material for: Sustainable Exploitation of Apple By-Products: A Retrospective Analysis of Pilot-Scale Extraction Tests Using Hydrodynamic Cavitation
Source: Foods. 2025 May 28;14(11):1915. doi: 10.3390/foods14111915 (PMC12154374; doi:10.3390/foods14111915)
Supplement: Supplementary file 1 [file foods-14-01915-s001.zip › foods-3627141-supplementary.pdf]

Article

Sustainable Exploitation of Apple By-Products: A Retrospective Analysis of Pilot-Scale Extraction Tests Using Hydrodynamic Cavitation

Luca Tagliavento [1,†], Tiziana Nardin [2,†,*], Jasmine Chini [3], Nicola Vighi [3], Luca Lovatti [4], Lara Testai [5], Francesco Meneguzzo [6,*], Roberto Larcher [2,††] and Federica Zabini [6,††]

1 HyRes S.r.l., via Salvator Rosa 18, 82100 Benevento, Italy; luca.tagliavento@hyres.it
2 Technological Transfer Center , FEM-IASMA Fondazione Edmund Mach, Istituto Agrario di San Michele all'Adige , Via E. Mach 1, San Michele all'Adige , 38010 Trento, Italy; roberto.larcher@fmach.it
3 Consorzio Melinda sca, Via Trento 200/9, 38023 Cles (TN); jasmine.chini@melinda.it (J.C.); nicola.vighi@melinda.it (N.V.)
4 CIF Consorzio Innovazione Frutta, Via Edmondo Mach 1, 38010 San Michele all'Adige (TN), Italy; lovatti@cif.tn.it
5 Department of Pharmacy, University of Pisa, 56126 Pisa, Italy; lara.testai@unipi.it
6 Institute of Bioeconomy, National Research Council of Italy, Via Madonna del Piano 10, 50019 Florence, Italy; federica.zabini@cnr.it (F.Z.)
* Correspondence: francesco.meneguzzo@cnr.it (F.M.); tiziana.nardin@fmach.it (T.N.); Tel.: +39-392-9850002 (F.M.); +39-329-6558658 (T.N.)
† These authors contributed equally to this work.
†† These authors contributed equally to this work.

Supplementary materials

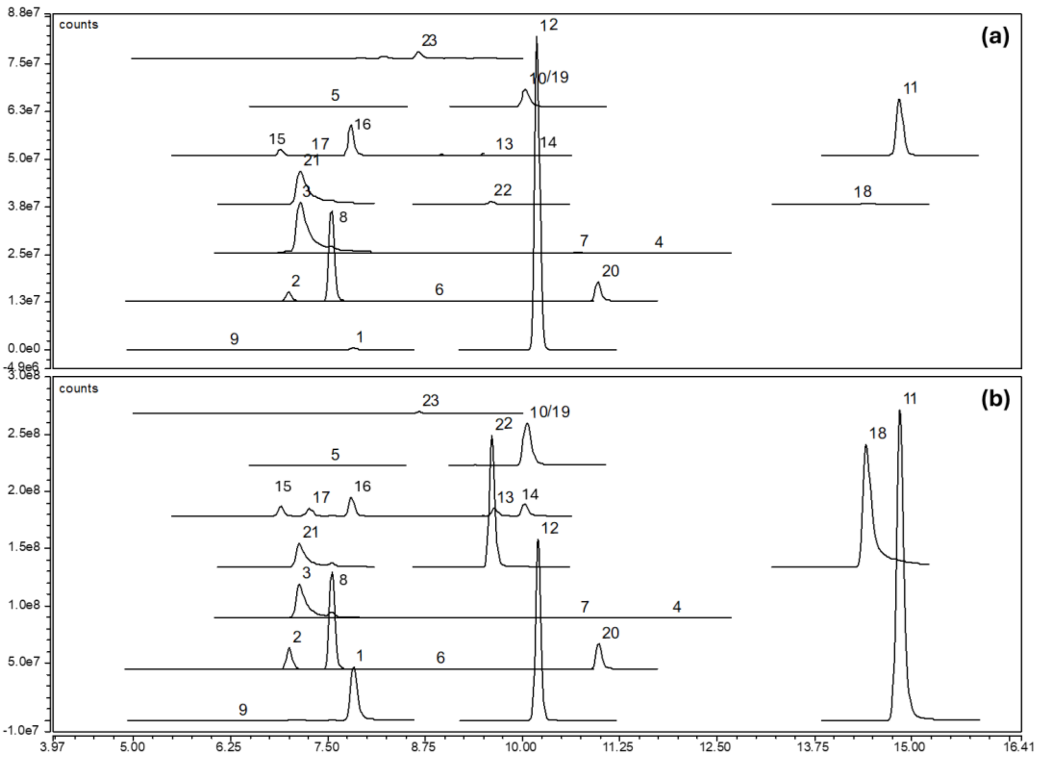

Figure S1. LC-HRMS chromatograms: (**a**) Apple pomace sample used in test REP4; (**b**) Apple pomace sample used in test REP4 spiked with a 2 mg/L available standards. 1=Caffeic acid; 2=Cathechin; 3=Chlorogenic acid; 4=Cinnamic acid; 5=Dehydrodicaffeoylquinic acid; 6=Dimers; 7=Di-O-caffeoylquinic acid; 7=Di-O-caffeoylquinic acid; 8=Epichatechin; 9=Glucosyl-quinic acid; 10=Hyperoside; 11=Phloretin; 12=Phlorizin; 13=Procyanidin A1; 14=Procyanidin A2; 15=Procyanidin B1;

16=Procyanidin B2; 17=Procyanidin B3; 18=Quercetin; 19=Quercetin-3-glucoside; 20=Quercitrin; 21=Quinic acid; 22=Rutin; 23=Trimers.

Table S1. Concentration of compounds reported in Table 2 for both AP samples and APE samples at the peak process yield. Data are reported in units of mg per kg dry weight of the raw biomass.

	ID Test									
	AP samples					APE samples				
**Compound**	**REW1**	**REP1**	**REP2**	**REP3**	**REP4**	**REW1**	**REP1**	**REP2**	**REP3**	**REP4**
Caffeic acid	0.3±0.2 ^{b}	1.71±0.2 ^{a}	0.33±0.02 ^{ab}	0.82±0.1 ^{ab}	1.16±0.1 ^{ab}	0.58±0.1 ^{ab}	1.09±0.1 ^{a}	0.24±0.1 ^{b}	0.66±0.1 ^{ab}	0.7±0.1 ^{ab}
Catechin	236±16 ^{a}	77.1±6 ^{ab}	87.6±8 ^{ab}	147±13 ^{ab}	21.5±2 ^{b}	218±16 ^{a}	76.4±5 ^{ab}	73.4±6 ^{ab}	127±12 ^{ab}	23.4±2 ^{b}
Chlorogenic acid	1712±119 ^{ab}	1628±158 ^{ab}	1627±140 ^{ab}	2209±201 ^{a}	658±42 ^{b}	1488±119 ^{a}	1622±159 ^{a}	1547±138 ^{a}	1474±151 ^{a}	644±51 ^{a}
Cinnamic acid	<0.1	<0.1	<0.1	<0.1	< 0.1	<0.1	<0.1	<0.1	<0.1	<0.1
Dehydrodicaffeoylquinic acids	112±8 ^{a}	18.6±1 ^{ab}	35.8±3 ^{ab}	25±2 ^{ab}	0.63±0.04 ^{b}	89.5±7 ^{a}	18.1±2 ^{ab}	35.1±3 ^{ab}	23.3±2 ^{ab}	<0.1 ^{b}
Dimers C-F	39±3 ^{a}	6.96±0.5 ^{ab}	7.19±0.6 ^{ab}	10±1 ^{ab}	1.06±0.1 ^{b}	37.5±3 ^{a}	6.6±0.5 ^{ab}	5.85±0.5 ^{ab}	10.1±1 ^{ab}	1.03±0.1 ^{b}
Di-O-caffeoylquinic acid	5.7±0.4 ^{ab}	18.6±2 ^{ab}	17.9±1 ^{ab}	25±2 ^{a}	9.1±0.6 ^{b}	5.12±0.4 ^{b}	16.5±1 ^{ab}	14.5±1 ^{ab}	24.5±2 ^{a}	7.25±0.5 ^{ab}
Epicatechin	672±47 ^{ab}	269±26 ^{b}	728±43 ^{a}	640±58 ^{ab}	325±21 ^{ab}	677±54 ^{a}	260±25 ^{b}	543±49 ^{ab}	348±32 ^{ab}	289±23 ^{ab}
Glucosyl-quinic acid	2.4±0.2 ^{ab}	6.91±0.5 ^{ab}	7.09±0.6 ^{ab}	9.79±0.8 ^{a}	< 0.1 ^{b}	1.98±0.2 ^{ab}	6.85±0.6 ^{ab}	5.83±0.5 ^{ab}	9.49±0.7 ^{a}	<0.1 ^{b}
Phloretin	0.3±0.02 ^{b}	6.6±0.6 ^{ab}	7.53±0.5 ^{a}	6.07±0.6 ^{ab}	4.86±0.3 ^{ab}	0.4±0.1 ^{b}	2.24±0.2 ^{ab}	1.23±0.1 ^{ab}	0.79±0.1 ^{ab}	4.85±0.5 ^{a}
Phlorizin	492±34 ^{a}	628±61 ^{a}	959±82 ^{a}	640±58 ^{a}	798±51 ^{a}	468±38 ^{a}	552±54 ^{a}	914±82 ^{a}	612±57 ^{a}	767±52 ^{a}
Procyanidin A 1	< 0.1 ^{b}	5.47±0.5 ^{ab}	2.96±0.3 ^{ab}	6.4±0.5 ^{a}	< 0.1 ^{b}	<0.1 ^{b}	5.81±0.5 ^{a}	2.92±0.2 ^{a}	6.31±0.5 ^{a}	<0.1 ^{b}
Procyanidin A 2	1.80±0.1 ^{ab}	13.4±1 ^{ab}	9.47±0.7 ^{ab}	16.3±1 ^{a}	< 0.1 ^{b}	11.4±0.9 ^{ab}	12.4±1 ^{ab}	8.76±0.7 ^{ab}	15.2±1 ^{a}	<0.1 ^{b}
Procyanidin B1	232±16 ^{a}	82±7 ^{ab}	95.9±8 ^{ab}	187±15 ^{ab}	51.5±4 ^{b}	189±15 ^{a}	75.7±6 ^{ab}	92.8±7 ^{ab}	149±10 ^{ab}	45.9±4 ^{b}
Procyanidin B2	918±64 ^{ab}	650±63 ^{ab}	858±74 ^{ab}	1298±118 ^{a}	314±20 ^{b}	846±68 ^{ab}	470±46 ^{ab}	829±85 ^{ab}	1254±116 ^{a}	303±24 ^{b}
Procyanidin B3	<0.1	<0.1	<0.1	<0.1	< 0.1	<0.1	<0.1	<0.1	<0.1	<0.1
Quercetin	<0.1 ^{b}	1±0.1 ^{ab}	1.18±0.1 ^{a}	0.76±0.1 ^{ab}	< 0.1 ^{b}	<0.1 ^{b}	<0.1 ^{b}	<0.1 ^{b}	0.7±0.1 ^{a}	<0.1 ^{b}
Quercetin-3-glucoside / Hyperoside	20±2 ^{b}	92.9±8 ^{ab}	50.8±4 ^{ab}	124±11 ^{a}	61.3±4 ^{ab}	24±2 ^{b}	81.6±6 ^{ab}	46.8±3 ^{ab}	120±11 ^{a}	53.5±4 ^{ab}
Quercitrin	26.1±2 ^{b}	59±4 ^{ab}	59.2±4 ^{ab}	72.7±7 ^{a}	53.9±5 ^{ab}	25.4±2 ^{b}	53.6±5 ^{ab}	46.1±4 ^{ab}	65.8±5 ^{a}	47.5±4 ^{ab}
Quinic acid	689±50 ^{a}	519±47 ^{ab}	428±30 ^{ab}	554±39 ^{ab}	185±15 ^{b}	593±44 ^{a}	368±26 ^{ab}	370±30 ^{ab}	494±44 ^{ab}	170±15 ^{b}
Rutin	35.8±2 ^{ab}	65.6±5 ^{ab}	77.9±7 ^{ab}	106±10 ^{a}	10.3±1 ^{b}	30.6±2 ^{ab}	62.1±5 ^{ab}	71.7±5 ^{ab}	94.2±7 ^{a}	8.46±0.8 ^{b}
Trimers C-C-C	261±20 ^{a}	30.4±3 ^{b}	39.2±3 ^{ab}	50.7±4 ^{ab}	64.1±5 ^{ab}	234±18 ^{a}	25±2 ^{ab}	37.4±3 ^{ab}	51.3±4 ^{ab}	73.2±5 ^{ab}
